# Experimental Models to Study End-Organ Morbidity in Sleep Apnea: Lessons Learned and Future Directions

**DOI:** 10.3390/ijms232214430

**Published:** 2022-11-20

**Authors:** Ramon Farré, Isaac Almendros, Miguel-Ángel Martínez-García, David Gozal

**Affiliations:** 1 Unitat de Biofísica i Bioenginyeria, Facultat de Medicina i Ciències de la Salut, Universitat de Barcelona, 08036 Barcelona, Spain; 2 CIBER de Enfermedades Respiratorias, 1964603 Madrid, Spain; 3 Institut Investigacions Biomediques August Pi Sunyer, 08036 Barcelona, Spain; 4 Pneumology Department, University and Polytechnic La Fe Hospital, 46026 Valencia, Spain; 5 Department of Child Health and Child Health Research Institute, School of Medicine, The University of Missouri, Columbia, MO 65201, USA

**Keywords:** intermittent hypoxia, airway obstruction, sleep fragmentation, cell model, animal model, human model, oxygen supplementation, CPAP withdrawal, sleep apnea pathophysiology

## Abstract

Sleep apnea (SA) is a very prevalent sleep breathing disorder mainly characterized by intermittent hypoxemia and sleep fragmentation, with ensuing systemic inflammation, oxidative stress, and immune deregulation. These perturbations promote the risk of end-organ morbidity, such that SA patients are at increased risk of cardiovascular, neurocognitive, metabolic and malignant disorders. Investigating the potential mechanisms underlying SA-induced end-organ dysfunction requires the use of comprehensive experimental models at the cell, animal and human levels. This review is primarily focused on the experimental models employed to date in the study of the consequences of SA and tackles 3 different approaches. First, cell culture systems whereby controlled patterns of intermittent hypoxia cycling fast enough to mimic the rates of episodic hypoxemia experienced by patients with SA. Second, animal models consisting of implementing realistic upper airway obstruction patterns, intermittent hypoxia, or sleep fragmentation such as to reproduce the noxious events characterizing SA. Finally, human SA models, which consist either in subjecting healthy volunteers to intermittent hypoxia or sleep fragmentation, or alternatively applying oxygen supplementation or temporary nasal pressure therapy withdrawal to SA patients. The advantages, limitations, and potential improvements of these models along with some of their pertinent findings are reviewed.

## 1. Introduction

Sleep apnea (SA) is a very prevalent disease affecting patients of both sexes and all ages, from infants to children to the elderly. Although SA was initially identified in Western countries and developed economies, the prevalence of SA is also remarkably elevated in developing countries and emerging economies [1]. Given that obesity and overweight are a major risk factor for this nocturnal respiratory disorder, the increasing worldwide trends denoting steady increases in the obesity epidemic-globesity-forecasts a parallel and steady increase in SA prevalence over the next several decades. SA is the result of abnormal collapsibility of the extrathoracic upper airway during sleep, resulting in recurrent airway obstructions (partial or total) leading to cyclic hypopneas and apneas, which can occur at rates sometimes exceeding 120 per hour of sleep. In general, the presence of less than 5 respiratory events per hour of sleep is considered as normal in adults, with severe SA consisting of >30 events/h [2]. These periodic apneas/hypopneas usually result in intermittent systemic hypoxemia affecting all patient’s tissues and organs and triggering activation of noxious cascades such as systemic inflammation and oxidative stress [3]. Moreover, the periodic arousals ensuing at the end of each obstructive event usually promote discontinuity of sleep and induce sleep fragmentation throughout the night. As a consequence of these biological perturbations, SA patients present symptoms such as diurnal somnolence, poor quality of life, depression and mood disturbances, and are at a greatly increased risk of traffic/labor accidents [4]. Moreover, SA induces mid-term and long-term end-organ morbidities usually manifesting as an increased risk of cardiovascular, metabolic, neurocognitive, and malignant diseases.

Due to the high prevalence of SA globally, nowadays an estimated 1 billion people being affected [1], and because there are independent associations of SA with such a long and important list of morbid consequences, a variety of experimental models have been developed and extensively used to investigate the systemic and end-organ consequences induced by SA. These models cover a wide scale—cells, animals, humans—and an ample spectrum of challenges being tested—recurrent airway occlusion, intermittent hypoxia/hypercapnia, and sleep fragmentation. Whereas in some cases the models are focused on exploring a specific effect on a particular cell type—e.g., oxidative stress in endothelial cells—, other models attempt to investigate the whole pathophysiological response in animals or healthy humans when subjected to injurious challenges realistically mimicking those experienced by patients with SA [5]. As in all cases of biomedical research on complex diseases, each scale of experimental approach has its advantages and disadvantages. On the one hand, very fine-tuned in vitro experiments focused on one pathway in one cell type allow researchers to dissect the question posed in a very precise way, but at the cost of neglecting the considerable number of physiological interactions arising from the multiorgan in vivo complexity of SA consequences. Conversely, SA models in animals or humans allow investigators to gain a more general perspective of the in vivo consequences of the specific challenge under study, but with a reduced capacity to dissect the pathophysiological role played by each cell type, tissue and organ involved. Therefore, a better understanding of the pathophysiology of SA consequences will usually require combining the information obtained from all different experimental approaches and scales. Although this intellectual exercise may seem relatively simple, the vast complexity of the pathophysiological processes in a disease involving multiorgan alterations has clearly hampered our ability to identify and modify the causal pathways underlying SA-associated morbidities [6]. Here, we describe the different cellular, animal, and human experimental models employed in research aiming to elucidate the consequences of SA, while also indicating their advantages, limitations, and potential improvements.

## 2. Cell Culture Models

The events of recurrent hypoxemia experienced by patients with SA are transmitted from the arterial circulation to the systemic capillary network and then to the surrounding tissues and cells. Given the different capillary densities of the various tissues, the heterogenous flow distribution patterns, and the divergent oxygen consumption in each tissue, the degree of hypoxia/reoxygenation in cells is not uniform among the various tissues/organs. For instance, aortic endothelial cells in SA patients are subjected to oxygen partial pressures swings ranging from normal values of 13% O_2_ to ≈4% O_2_, corresponding to 100 mmHg and ≈30 mmHg (SaO_2_ nadir of 60% in severe SA). In contrast, cells in other tissues experience lower oxygen tension levels and smaller amplitude swings, which have been measured by using O_2_ microsensors in animals subjected to intermittent hypoxia mimicking SA, e.g., ≈2.5–5% (≈20–35 mmHg) swings in the brain or peripheral muscle [7,8,9,10]. This fact has usually been underappreciated in cell culture experiments, but current optimized settings allowing for precise control of culture cell oxygenation reveal its importance in SA research. For instance, it has been shown that wound healing experiments involving human aortic endothelial cells considerably depends on the hypoxic regimen imposed: 1–20% (usual setting in IH cell culture research) or 4–13% O_2_ (representative in arterial blood in SA) at frequencies of 0.6, 6 and 60 cycles/h) [11]. Moreover, the proliferation of human lung cancer cells may substantially differ when such cells are subjected to IH (60 cycles/h) when the oscillations in oxygen tension ranged from 7–13% O_2_ or 4–7% O_2_, i.e., mimicking only SA or SA overlapping with COPD [12]. Hence, the limited data available to date point out to the need for culture settings to be set such as to reproduce, as best as possible, the actual hypoxia/reoxygenation events experienced by cells in the different tissues perfused by arterial blood with typical SaO_2_ swings in SA patients.

### 2.1. Conventional Cell Culture Models of Intermittent Hypoxia

Unfortunately, the conventional cell culture setting can hardly, if at all fulfill the requirements to realistically mimic the IH oscillations typically encountered in SA [13]. Indeed, in addition to the inherent difficulties associated with accurately defining cell oxygenation under static conditions [13,14], IH paradigms for cell culture in the context of SA research impose the added requirement of cycling cell oxygenation at a very high rate (up to 60 cycles/h in severe SA). The conventional setting to apply IH in cultured cells is based on cyclically changing the oxygen content of the air above the cell culture medium and thus assuming that cells cultured at the bottom of the plate are subjected to the same de-oxygenation and oxygenation cycles. Nevertheless, this assumption is questioned by two main facts. First, even under static conditions (i.e., keeping constant the O_2_ concentration in the air above the cell culture), it is difficult to ensure that all cells within a given plate will experience such a level of oxygenation. The reason is that cells consume oxygen and hence there is a vertical gradient of O_2_ concentration from cell level to the top surface of the cell culture medium. In fact, the actual oxygen concentration depends on the number of cells cultured and on their O_2_ consumption rate [15]. In addition, circulation of the culture medium caused by spontaneous passive convection induces a horizontal gradient of gas concentration at the bottom of the culture dish and thus at the cell level [16]. In addition, another more relevant issue makes it difficult to subject cultured cells to well-controlled IH in the conventional cell culture setting in which passive diffusion gas exchange is implemented. Indeed, the mechanism by which a change of O_2_ concentration in the air above the cell culture is transmitted to the cells at the bottom of the plate is by gas diffusion within a liquid. However, this passive process is very slow as compared with the frequencies of IH in SA. The time required to fully equilibrate the medium with the external oxygen concentration has been measured and can require more than one hour [15]. As a result, the maximum rate of IH achieved at the cell level with any conventional setting is of a few cycles per hour [17,18], quite lower than rates observed in moderate and severe SA (30–60 events/h). Since it is difficult to predict what is the actual O_2_ partial pressure at the level of the cultured cell microenvironment in any given IH experiment, it could be of interest and certainly informative to measure it by means of small O_2_ sensors at the cell level [17,18,19]. However, this is clearly not a feasible approach for routine experiments, such that substantial degrees of variability in the actual magnitude and frequency of IH may occur unbeknownst to the experimenters.

### 2.2. Improved Cell Culture Models for Intermittent Hypoxia

**Figure 1:**
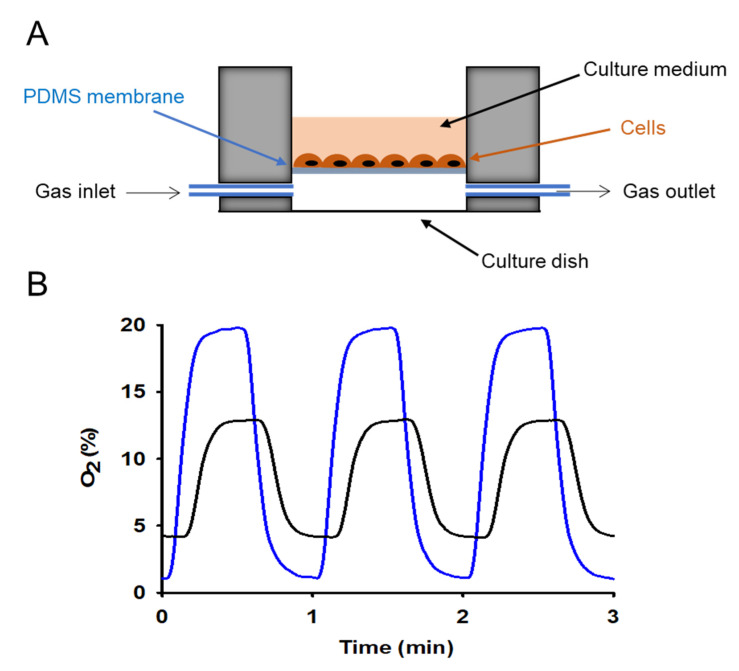
Experimental in vitro system for cellular exposures to constant or variable oxygen concentrations. (**A**) schematic view of the cell culture setting to apply controlled intermittent hypoxia (IH) to cultured cells (see text for explanation). (**B**) actual oxygen concentration measured on top of the membrane (cell culture level) when applying IH with different magnitudes (1–20% O_2_ (blue line) and 4–13% O_2_ (black line) at a frequency of 60 cycles/h. Reprinted with permission from Ref. [11]. 2017. American Physiological Society.

Different alternative cell culture settings have been proposed for IH research in SA. Instead of relying on passive diffusion, they are based on the convection of the culture medium. In one case, employing a setting in which cells were cultured inside capillary tubes and forced media convection allowed to apply a pattern of intermittent hypoxia ranging ≈0–80 mmHg of O_2_ partial pressure with a change time constant of less than 2 s [20]. In a different approach proposal, peristaltic pumps were used to alternatively subject cells to a culture medium from two containers, one flushed with normoxic gas and the other one flushed and saturated at the desired level of hypoxia. This setting was able to control O_2_ concentration in the cell microenvironment at rates of up to 60 cycles/h [19]. Nevertheless, settings based on cell medium convection are not optimal since the moving medium imposes cell shear stress which is a second hit added to IH, thus a potentially confusing factor particularly important in cells with sensitivity responses to mechanical stimuli (e.g., blood vessel-derived cells and tumor cells) [21,22]. Accordingly, medium convection-based settings have been scarcely used and have been somewhat abandoned in IH cell culture research. There is another alternative to the conventional cell culture setting which has been developed more recently and has shown to be particularly well suited for SA research in cells. It simply consists of replacing the glass/plastic bottom of the cell culture well with a thin membrane of polydimethylsiloxane (PDMS) which is a biocompatible type of silicone with an excellent coefficient of diffusion for O_2_ [13]. For instance, a PDMS membrane with a thickness of 100 µm has an O_2_ diffusion time of ≈0.5 s. Hence, circulating air with O_2_ concentration cycling at the stipulated frequencies mimicking SA subjects the cultured cells to a well-controlled pattern of IH. As PDMS is transparent, the setting is compatible with microscopy techniques (Figure 1). This approach based on thin permeable membranes has been used in different IH settings either on customized or commercially available gas-permeable culture plates [11,23,24,25,26,27,28]. It has already provided important initial findings further demonstrating the relevance of precisely controlling IH in SA research with cell cultures [11,12].

### 2.3. Future Directions: Intermittent Hypoxia Models in 3D-Cultured Cells

The optimized 2D cell culture models described above are well suited for testing the effect of IH on cell monolayers, thus in cells naturally living in this 2D geometrical configuration such as endothelial and epithelial cells. However, in vivo, most cells are normally residing in 3D tissues in an environment shared by other cells embedded into an extracellular matrix, both in normal tissues, in cancer tumors, and in experimental organoids. Therefore, to physiomimetically test the effects of IH, a 3D cell culture model is required. Fortunately, recent developments facilitate the process of 3D culturing cells within hydrogels embedded in bioprinted constructs with/out microchannels for perfusion [29,30,31,32]. However, controlling the magnitude of IH is not simple given the finite diffusion capacity within 3D constructs. Recent proposals of microchips for 3D cell culture within hydrogels made from lung extracellular matrix suggest that the application of controlled patterns of intermittent hypoxia to adequately mimic SA could be possible [33], but more work is still required to design and characterize such experimental systems, and to simplify their construction and assembly procedures so that they can be easily available for most cell biology laboratories.

## 3. Animal Models

This review addresses experimental models of SA focused on investigating the end-organ consequences of the disease [34,35]. Specifically, models challenging the physiological system (either at the cellular, tissue/organ or whole-body level) with the main noxious stimuli that patients experience owing to the respiratory events in SA: recurrent airway obstruction, intermittent hypoxia/hypercapnia and sleep fragmentation. Accordingly, naturally occurring SA (e.g., in specific breeds of dogs and pig) [36,37] will not be taken into consideration.

Subjecting a research animal to chronic recurrent obstructions at the airway opening during natural sleep is the ideal model to study the consequences of SA. Indeed, this model almost perfectly mimics the challenges experienced by SA patients during nocturnal apneas. These recurrent airway occlusions would induce associated events of hypoxia and hypercapnia, would increase intrathoracic swings of negative pressure as the animal tries to breathe in the context of a high resistive load or occluded airway and, importantly, such imposed events would elicit microarousals. Although such a model is feasible in big [38,39,40,41] and small animals [42,43,44,45,46], it is considerably complex and thus difficult to apply for extensive research involving large numbers of animals in chronic settings.

Therefore, investigating the consequences of SA is usually carried out in rodents with settings that separately apply the different challenges: recurrent airway obstructions, intermittent hypoxia/hypercapnia, or sleep fragmentation.

### 3.1. Airway Obstruction Models

Early investigators in the field of experimental SA had already realized the importance of airway obstructions and therefore developed and implemented the animal SA model which is conceptually the most suitable for investigating the consequences of the disease. The model requires surgically modifying the trachea to place an occlusion valve or creating a permanent tracheostomy to occlude the trachea with an inflatable balloon and placing EEG electrodes to monitor sleep. Realistic recurrent airway obstructions can then be applied precisely when the animal is spontaneously sleeping as monitored by the EEG. Remarkably, in a study where the setting was chronically applied for several months in dogs, the investigators demonstrated that this realistic model of SA induces hypertension [38]. Other applications of this type of model of airway obstruction were mainly focused on studying the effects of the long-standing airway occlusions challenge on hemodynamics and the cardiovascular system [39,40,41,47]. In one of these studies, the authors assessed occlusion-induced hypertension depending on whether apneas were finished by an arousal or not. They showed that arterial blood pressure was considerably higher when arousal occurred, thereby concluding that this sleep alteration produces a separate, additional acute hypertensive response [47]. Although it is conceptually ideal for mimicking SA, the model for applying airway obstruction synchronized with natural sleep is very technically and maintenance demanding and its application has progressively subsided. More recently, a new model for the chronic application of airway obstructions in freely moving rats has been described [42]. The setting is based on the permanent implantation of an inflatable obstruction device in the trachea and on applying airway obstructions (selectively at end-inspiration or end-expiration). This setting can obstruct the airway at a rate of up to 60 times/h (each apnea lasting 10 s) for 8 h during the sleep cycle for up to 4 weeks [42,48]. This model has the interesting advantage of applying realistic apneas by airway occlusions in rats, which partially reduces logistic requirements compared to previous settings in big-size species, but with the inherent limitation that obstructions are not necessarily synchronized with sleep. Following a completely different approach, it has been proposed to realistically induce airway obstructions mimicking SA by artificially increasing the collapsibility of the upper airway of healthy animals to spontaneously experience SA events. For instance, injection of liquid collagen in the uvula, tongue, and pharyngeal walls of monkeys induced hypopneas [49]. Using the rabbit as a model, other authors have injected saline into the tongue base, botulinum toxin type A into the genioglossus or polyacrylamide gel in the submucous muscular layer of the soft palate [50,51,52]. Other approaches have been proposed pursuing active or passive alteration of the collapsibility of the upper airway [43,44,45]. Although these models have been shown to induce patterns of SA events, they have not been thoroughly characterized and are not widely used for investigating the end-organ consequences of the disease.

**Figure 2:**
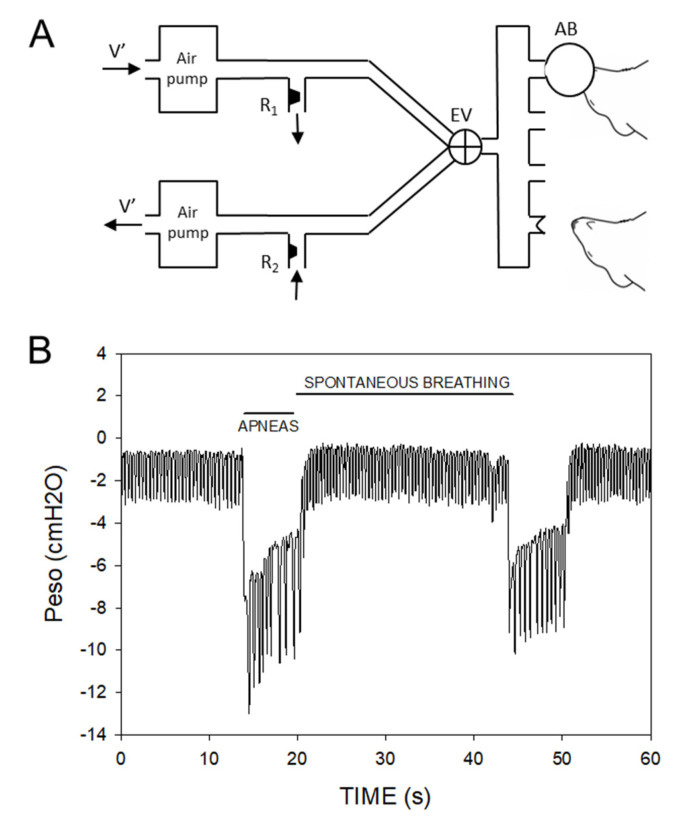
Model to apply airway obstruction mimicking sleep apnea. (**A**) Diagram of the system. V′: flow generated by both air pumps (reversed directions). R1 and R2: resistors. EV: three-way electrovalve. The resistance of R1 was greater than that of R2 to generate higher positive (30 cmH_2_O) than negative (−5 cmH_2_O) pressure. Four airbags were connected to the source of alternant pressure. For the sake of simplicity and illustration, the figure shows only two airbags in place: one inflated (30 cmH_2_O) to apply airway obstruction to the mouse (top) and the other one deflated (−5 cmH_2_O) to allow the mouse to breathe spontaneously. (**B**) Example of esophageal pressure (Peso) recording in one mouse during application of two of apneas (6 s each at a rate of 120/h). Reprinted with permission from Ref. [55]. 2011. Elsevier.

More simplified settings have been employed to investigate the effects of airway obstructions in SA. Some of them are invasive (tracheal intubation or application of a collapsible airway segment) and require anesthesia, which enables them for application exclusively in acute settings. Other models that are not invasive but still require anesthesia have been proposed [53,54]. In one case, a specially designed mask providing either airway obstruction or only IH has been proposed. This setting allowed to document that oxygenation in different body tissues depends on whether oxygen desaturations are accompanied or not by airway obstructions, thereby confirming the interest in investigating both airway obstruction and IH challenges [7]. In another instance, airway obstructions were applied by means of a computer-controlled air bag system, showing that the setting was able to induce variability patterns on desaturation cycles similar to the ones observed in patients (Figure 2) [55]. However, since these settings require anesthesia or at least sedation, their application for acute settings is straightforward but whether they are applicable chronically is unclear. To avoid anesthesia, another model consists of placing a rat in a setting with two chambers separated by an adjustable neck collar: one chamber is for the head and the other one is a restrainer for the rest of the body. The head chamber has an orifice to allow breathing with a valve that imposes recurrent occlusions (e.g., 5-s, 60 cycles/h) [56]. The potential stress induced by animal restraints was found to be minor after a few sessions of training. This setting has been applied to investigate acute and chronic effects of airway obstructions mimicking SA in the cardiovascular system [57,58,59].

### 3.2. Intermittent Hypoxia/Hypercapnia Models

**Figure 3:**
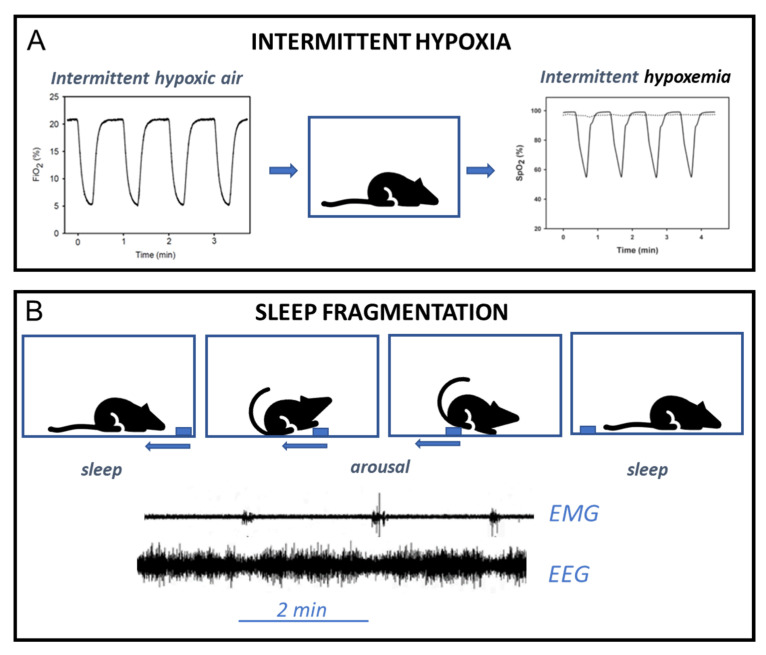
Diagrams of the most common experimental animal models of sleep apnea. (**A**) Application of intermittent hypoxia: air with cyclic O_2_ fraction (FiO_2_) in the animal cage induces recurrent hypoxemia (SaO_2_). (**B**) Induction of sleep fragmentation: smooth cyclic movement of a bar (blue) in the cage ground induces cyclic arousal, as reflected by electromyography (EMG) and electroencephalography (EEG) signals in the mice. See text for explanation. Reprinted from Ref. [6]. Creative Commons License.

Models of hypoxia/hypercapnia are aimed at subjecting animals to exclusively one of the main injurious challenges experienced by patients with SA resulting from apneas/hypopneas: recurrent alterations in blood gases, namely hypoxia, and hypercapnia. These models, so far mainly focused on hypoxia, are based on inducing intermittent changes in blood gases by cyclically modifying the gas composition of the air breathed by the animals (Figure 3). The first chronic SA model of IH consisted of transiently reducing O_2_ concentration to 3–5% for 3–6 s twice per minute (6–8 h daily for 35 days) and was used to prove that this SA-mimicking challenge induced arterial hypertension and left ventricular hypertrophy [60]. Implementation of this type of setting is straightforward since the application of IH only requires a valve-controlled system able to alternatively inject room air of nitrogen into the cage so that the composition of the air breathed by the animal changes progressively with a time profile similar to the one observed in patients. In the case of IH-hypercapnia, nitrogen is enriched with CO_2_ to achieve the desired level of hypercapnia. It should be mentioned that the addition of simultaneous intermittent hypercapnia to IH for better mimicking the clinical situation in SA was started early [61] although with a relatively low number of accumulated publications but with sustained interest [62,63,64,65,66]. The IH model is very flexible since by controlling the timing of hypoxic gas injection, it is possible to induce selective changes in the SaO_2_ of both the hypoxic and reoxygenation phases of each cycle [67]. Moreover, it is possible to model the whole-night variability in the hypoxia-reoxygenation cycles observed in patients [68]. As the IH model can be implemented in the cages where the animals normally live, the hypoxic/hypercapnic challenge can be applied automatically with almost no other alterations in the animal’s living conditions and, importantly can be applied chronically for several weeks up to six months [69]. It is important to note that the experimental setting requires careful implementation to achieve uniform change in gas concentrations within the cage, with low operating noise to minimally disturb the animal environment. Indeed, given that mimicking high rates of hypoxia/reoxygenation (up to 60 events/h as in severe SA) allows a short time (and hence requires high flow) to modify the gas composition in the cage, the set design (e.g., cage volume, injecting flow, geometry of gas injecting orifices) should be optimized [70].

However, some limitations of the IH model should be taken into consideration. The first one concerns the basic hypothesis that changing the gas breathed by the animal only produces alterations in blood gases. In fact, it has been shown that some sleep disturbances are elicited by IH [71,72,73,74]. Specifically, it was reported that in case the hypoxic episode was started during sleep, arousal appeared always at hypoxic nadir [72,74], with global alterations in both REM and non-REM sleep that persisted at least for 3–5 days of IH application. Nevertheless, sleep alterations induced by long-term (8-week) IH persisted for at least 2 more weeks [73,74]. Interestingly, such sleep side effects of IH are in the line of partially reproducing the sleep fragmentation experienced by SA patients during the apneic events, and therefore could partially contribute to realistically mimicking the challenges suffered by patients. However, interpretation of the results from IH experiments only in terms of hypoxia-reoxygenation should be carefully done since the potential effects of the associated sleep alterations in each experiment are unknown. The second limitation of the IH model is that animals subjected to IH may experience other alterations that make difficult the interpretation of data in terms of IH exclusively. For instance, IH induces changes in food ingestive behaviors and in body weight as well as promoting the emergence of gut microbiota dysbiosis [10,66,75]. Whether these alterations in gut microbiome are a consequence of metabolic changes induced by tissue/organ hypoxia-reoxygenation or a side effect of experimentally induced stress remains unclear. A third limitation of the conventional IH model is the fact that IH is applied according to the timing set by the experimental equipment in a way independent of animal sleep status. Whereas hypoxemic events in SA patients are synchronized with arousals, IH in the conventional model is applied during the light period, i.e., rodent preferential sleep time, but regardless of whether the animal is actually sleeping or awake. To improve the model for ensuring that IH is applied only during sleep, a refinement of the setting includes mouse sleep monitoring so that IH is triggered only in case the animal is not awake [76,77]. However, the setting is unavoidably complex because of individual sleep monitoring and thus is difficult to apply in routine research, particularly in long-term chronic models. A fourth limitation of the IH model is that it does not allow us to determine whether the IH effects are caused because the cells in a tissue are directly sensitive to the low oxygen pressure in their local hypoxic microenvironment or because they are exposed to the influence of circulating factors systemically secreted in response to hypoxia. Interestingly, a parabiotic model can be useful for distinguishing between the effects of local and systemic hypoxia [78]. According to this setting, one of the parabionts is normally oxygenated while simultaneously exposed to the whole systemic response induced by intermittent hypoxia in his/her parabiont.

Although not a limitation of the model, there is an open question on the definition of the IH paradigm that should be taken into consideration in terms of clinical translation of the results. For instance, what are the most realistic frequencies and magnitude of the hypoxic events applied to animals for mimicking SA patients is not clear, because of the considerable inter-species differences between humans and rodents (which are the almost exclusively used animals in IH research). Regarding the timing of hypoxia-reoxygenation, two viewpoints are possible. On the one hand, the duration of hypoxic events applied to animals should be in proportion to the animal breathing rate: whereas 15 s of hypoxia includes only a few human breathing cycles, this time corresponds to a high number of breaths in rats and even more in mice. According to this perspective, the duration of hypoxic cycles should be considerably reduced in rodents. On the other hand, if the relevant issue is the timing of hypoxia-reoxygenation at the tissue/cell level, the duration of hypoxic events applied to animals should be similar to in humans. A similar question arises regarding the severity of each of the hypoxic events applied to rodents. Specifically, considering that the oxygen dissociation curves are very different in human and rodent blood [79], the question is whether the IH nadir should be focused on achieving similar arterial oxygen desaturations as in patients or should be focused on terms of the associated oxygen partial pressure [79,80]. However, it should be mentioned that the results obtained with different IH paradigms are usually consistent and, when specifically tested, show a dose response [81,82,83,84]. Notwithstanding its limitations and open questions, the IH model (with/out intermittent hypercapnia) is widely accepted and has provided very useful information on the role that blood gas alterations play in the mechanisms inducing systemic and end-organ consequences of SA.

### 3.3. Sleep Fragmentation Models

An SF challenge mimicking the recurrent arousals experienced by patients with SA should be able to apply a short, minimally disturbing stimulus (the aim is to induce microarousals) which is repeated at a frequency similar to the apnea-hypopnea index in SA (15–60 events/h). To this end, the experimental settings already used for studying full sleep restriction—based on placing the animal on a small surface platform elevated on a water surface so that each time the animal enters sleep fells into the water—are not suitable since arousals are long (the animal must swim to get the dry platform) and sleep is interrupted each time the animal enters sleep. Different experimental settings have been devised for applying an SF challenge that approaches the pattern in SA patients in rodents. The setting can be designed to apply individualized arousals to each animal, for activating a miniature vibration motor mounted on a rat head [46], or to induce arousals to all the animals in the same cage. For instance, in a setting that was devised to apply sleep disturbances to simulate the recurrent awakenings of patients in ICUs, the cage of the animal was subjected to mechanical vibration (100 rpm, 20 s) every two minutes (i.e., 30 events/h) [85]. Other two settings followed a different approach based on short tactile stimulation: to produce a silent smooth movement in the floor of the cage forcing the animal to transiently move and hence experience short arousal. In one case, the animal is placed into a cylindrical cage that is divided into two separate parts and that has a floor that may rotate at the frequency defined by the investigator. As the disc rotates more than 180 degrees, the animal is aroused as it is forced to move to avoid the chamber divider. This setting has been used to apply SF at 30 cycles/s during the light (sleep) phase of the day for 4 and 9 consecutive days [86,87]. In another case, sleep arousals are induced by a modified treadmill [88] or a mechanical near silent motor with a horizontal bar sweeping just above the cage floor from one side to the other side in the standard mouse laboratory cage (Figure 3), which is commercially available and has been validated [89,90] for studying the SA consequences induced by SF (typically at 30 arousal/h).

Tactile-based devices to induce SF have the advantage that can be applied automatically and simultaneously to several animals in a cage, with no human intervention, hence minimizing the stress on the animal. Howeverchronologicaltwo potential caveats can be mentioned. First, although very short, the physical activity associated with arousals is at variance with arousal in SA patients, and the potential effect of this variance is unknown. Second, in common with the conventional IH setting, SF is applied during the theoretical sleep phase of the day but irrespective of the animal sleep stage. This problem could be avoided as it was proposed for solving the issue in IH, but again the need for individually monitoring sleep and SF application would make the setting unpractical for extensive research, particularly in chronic models. It should be mentioned that a procedure based on optogenetics—using light to activate genetically targeted neurons expressing a light-sensitive receptor—can be used to apply SF during sleep time and with arousals not involving movement [91,92,93]. This technology can provide interesting insights into sleep/arousal mechanisms but, given that the intervention should be applied individually on animals with the brain instrumented with laser fibers, its application for routine studies on the SA consequences elicited by chronic SF is considerably difficult and inordinately demanding as compared with the conventional SF settings.

### 3.4. Future Directions for Improving Animal Model Research

Achieving relevant translational conclusions on the pathophysiology of a complex disease by means of animal research mainly depends on the suitability of the models employed. Regarding the multi-organ consequences of SA, each of the main different models (recurrent airway obstructions, intermittent hypoxia/hypercapnia, and sleep fragmentation) has its own advantages and limitations, allowing us to evaluate to what extent the experimental results can be translated to the clinical problem. However, it is interesting to note that some of the limitations of the data from animal research do not arise only from the limitations of the specific models per se but also depend on the way in which the research is devised and performed. Indeed, as it will be mentioned below, with the aim of simplifying and somehow standardizing the protocols and experiments for better comparison with other literature data, some well-known important experimental factors can be overlooked. In that case, the derived conclusions—which are correct for the specific experiment—may lead to misinterpretation when extrapolated to the general context of the disease. This problem, which is common and not specific for animal research of SA consequences, is however particularly important in this chronic disease involving the physiological interaction of almost all organs. Indeed, although scarce, there is enough information to substantiate the potential relevance of this question regarding SA, thereby prompting us to improve the real-life implementation of animal research even when using the currently available models.

The sex of the animals employed is a most relevant open issue. The fact that SA and its consequences show different traits in men and women is well known from clinical research. The need to carry out research in animals of both sexes is so important, regardless of the research field, that the issue has been included in strong recommendations at institutional level [94]. However, research on the consequences of SA has been so far carried out almost exclusively on male animals -with the obvious exception of postmenopausal [95,96] and gestational SA models [90,91,97,98]. Specifically, IH elicits differential male and female responses in the susceptibility to oxidative injury and sleepiness [99], neural remodeling [100], metabolic response [101], protein expression of the vascular wall [102], respiratory-sympathetic coupling [103], or hypertensive response [104]. Moreover, the role that male and female sex hormones play in the response to IH has been reported by gonadectomy experiments in both sexes [105,106]. Notably, there is a transgenerational sex difference in the metabolic and epigenetic changes induced by gestational IH [107].

Another relevant issue in SA experimental research, which almost always is overlooked, is the age of the animals under challenge. Indeed, whereas SA prevalence increases with patient age, being relevant in the elder, almost all animals employed in SA research have been carried out in young rodents with an age equivalent to human late adolescence or very young adulthood: e.g., mice ≈2 month-old instead of ≈18–20 month-old (corresponding to human ≈20 year-old and 55–65 year-old, respectively [108]. This fact is relevant for a chronic disease such as SA as indicated by the few data available specifically comparing the effects of animal age. For instance, when subjected to IH, young and aged animals exhibit differences in susceptibility to biological injury [109], brain tissue hypoxia and oxidative stress [110], sexual response [106], cancer growth [111], or in cardiovascular remodeling [112,113]. Interestingly, the already few reported findings systematically indicate that, for a wide range of injury types, the deleterious effects of IH are more pronounced in young than in mature/elder animals. Accordingly, this information should be taken into consideration when translating the conclusions derived from (almost exclusively young animals) to SA patients, most of whom have greater chronological and biological age. Despite obvious potential confusion factors such as animal sex and age, other issues should be considered when investigating the consequences of SA. In particular, regarding several questions that sometimes are considered just minor technical details in the experimental design and thus overlooked.

The selection of the animal strain is one of these major issues. Indeed, the few data available indicate, for instance, that the changes induced by IH in the metabolic and inflammatory response [114], vascular remodeling and ET-1 expression [115], oxidative stress, and hormone responses [116] depend on the animal strain within each species. Remarkably, such differences may appear between very close sub-strains (e.g., C57BL/6N and C57BL/6J) [114]. It is also important to consider that laboratory animals, which are the result of homogeneous genetic breeding, present an immunological response considerably different from the one observed in wild animals [117]. In the wild mouse, the immune system cells are more readily activated than in lab strains and have a population of highly activated myeloid cells which are not present in laboratory mice [117]. This suggests that the inflammatory responses observed in laboratory strain mice following IH or SF could be reduced than if the challenges were applied to wild animals. Considering the relevance of inflammatory pathways in modulating SA consequences, these data are of potential interest from a translational viewpoint. To address this issue, possible naturalization approaches have been recently suggested [118,119,120].

Another important issue to consider is the social interaction among experimental animals. Whereas some conventional IH and SF settings allow the animals to live with their social group in the same cage, advanced variants of such settings (e.g., synchronizing IH to sleep or optogenetic application of SF) may require the animals to be isolated in individual cages. Given that isolation-induced stress may affect the immune response of the animal and increase cardiovascular alterations [121], memory impairment [122], and cancer progression [123], and that isolation housing conditions actually modulate the response to intermittent hypoxia [116], it seems that normal social interactions among animals should be preserved as much as possible, particularly in chronic settings in which the immune response may play a more relevant role.

Diet and activity under conventional housing conditions may also be particularly relevant. It has been shown that “control” laboratory rodents are metabolically morbid because of free access to food and limited opportunities to exercise [117]. Whereas such overfed sedentary control animals can be a good model for overweight and sedentary humans, they may be unsuitable for representing humans with normal weight and physical activity [124]. As chronic models of IH and SF show that these challenges induce metabolic changes, setting a realistic regime of food and exercise in healthy control animals would result in data with more translational interest.

Setting a physiologically reasonable ambient temperature may modulate the effects observed in SA models. This fact is important since the animal facility temperature set by local regulations does not necessarily coincide with thermoneutral temperature in rodents, being this fact a potentially confusing factor [125,126]. On the one hand, a difference of very few degrees may have an impact on baseline unchallenged animals (e.g., sleep and cardiovascular regulation [127] and cancer progression [128], both very relevant consequences of SA) and on how is the animal metabolic response to the IH challenge [129].

In addition to those well-known questions, it has been recently realized that gut microbiota plays a substantial role in the homeostasis of almost all body organs and systems, with gut microbiota alterations also observed in SA patients [130,131]. Therefore, control of animal microbiota is key in rodent models [132,133]. This fact may be relevant in SA research [134] since it has been shown that both IH [10,135,136] and SF [137] modify the gut microbiota in rodents. Given that, as compared with laboratory mice, wild mouse gut microbiota improves the resistance to diseases such as infection and cancer [138], it has recently been suggested that laboratory mice born to wild mice exhibit two advantages: they model human immune responses and have natural mouse microbiota [139]. Future studies subjecting this type of animals to IH and SF would be extremely useful for improving the translation of results from the lab to SA patients.

The apparently minor experimental issues previously mentioned may considerably contribute to the devilish problem of experimental data variability [140]. In this context, it is interesting to consider whether the propSAls trying to improve animal research by replacing the conventional setting with one closely mimicking the physiological animal environment [141,142] is applicable to the research of end-organ consequences of SA, for instance by applying IH and SF to animals living in more physiological conditions [143]. Interestingly, in addition to improving the experimental settings, deriving solid conclusions from research in animal models of SA may benefit from systematic reviews and meta-analyses (SRMAs) [144]. Indeed, recent SRMAs focused on IH-related alterations in vascular structure and function [145] and on the cardiac consequences of IH [146] have provided deeper insights than conventional narrative reviews. As recently suggested [144], future application of the individual participant data meta-analysis perspective [147] to research in animal models of SA may be particularly fruitful.

## 4. Human Models of SA

Human SA models are of high conceptual interest but unfortunately may be difficult to implement logistically and in some cases also ethically, hence reducing their potential applicability. Otherwise, most experimental tests so far carried out in animal models could have been performed in humans. Indeed, the usual intervention in SA models is simply to apply IH or SF and the outcome is to study a series of physiological or behavioral variables and biochemical, cellular, and structural tissue parameters, which in most cases are more easily obtained and processed in humans than in animals taking into account all the clinical and analytical tools available for detecting human pathologies. In addition to logistical and ethical difficulties, the main limitation of human SA models is regarding chronicity: whereas an IH/SF challenge applied for 4–8 weeks can be acceptably considered as chronic for mice given their life span, establishing a chronic SA model in humans would require a completely impractical experiment duration.

When weighing the pros and cons in human models of SA it is interesting to also consider biological variability, particularly in comparison with animal model research. Indeed, laboratory animals are very homogeneous both genotypically and phenotypically. This fact has the advantage of reducing biological variability and hence facilitating the extraction of clearer conclusions, but at the cost that the clinical translation of derived conclusions is limited by the very specific geno/phenotypes investigated and pathophysiological inter-species differences. By contrast, human models may provide conclusions covering a wide spectrum of population geno/phenotypes with direct clinical translation. However, achieving such conclusions would require a high number of subjects.

### 4.1. Experimental SA Models in Healthy Volunteers

Healthy human-based models of SA—i.e., subjecting volunteers to the specific challenges that the disease imposes on patients—are of considerable interest. Their use provides data on the integrated physiological response to specific SA challenges in humans, thereby avoiding the potential bias owing to interspecies differences when using animal models. Additionally, human SA models can cover an ample real-life variability of genotypes and phenotypes. However, the basic principle of the model set, either based on IH or SF, is the same in both animal and human models. Therefore, in this regard human and animal models exhibit the same advantages and limitations resulting from applying only one single challenge.

Most experimental human models of SA have focused on investigating the effects of both acute and long-term (from a few hours up to 4 weeks) IH challenges [148,149]. It should be mentioned that, as in the case of IH animal models, human SA models of IH are specifically different from those in the literature in which IH is applied for studying the effects of adaptation to cyclic altitude changes, exercise training or hypoxic preconditioning. In those studies, the duration of hypoxia/reoxygenation phases may last several minutes or more, by far much longer than in SA. The application of IH for mimicking SA in healthy volunteers is also based on inducing recurrent hypoxemia by cyclically breathing air with different oxygen concentrations. To this end, two technical procedures have been employed. In one of them, the subject wears a nasal/face mask which is connected to a valve system alternatively providing room air or hypoxic air. The severity of the IH challenge is determined by the duration of the hypoxic and recovery phases in each cycle (thus determining the frequency of events) and by the O_2_ concentration of the gas breathed during the hypoxic phase. These parameters can be individually tailored to achieve the desired values of nadir and recovery SaO_2_ which is continuously measured by pulse oximetry. This setting has the advantage that it is relatively easy to implement since is very similar to the one in a conventional sleep lab for CPAP titration during polysomnography. The only difference is that the patient’s mask is not connected to a CPAP device but to a setting providing cycling air. This approach has been used to mimic different apnea-hypopnea indices in patients: 60 cycles/h, corresponding to severe SA [150], 30 cycles/h as in intermediate SA [151,152,153,154,155,156,157,158,159], and 15 events/h as in mild SA [160,161,162]. It is worth noting that this type of mask-setting has been also applied to investigate the effects of intermittent hypercapnia (realistic cycles of 30 s of hypercapnic hypoxia followed by 2 min of air, i.e., 24 events/h)) in healthy humans [163]. Interestingly, this human model has recently been used to investigate whether the effects of intermittent hypoxia/hypercapnia are different in men and women [164,165]. A second possible procedure for applying IH to humans is different only from a practical perspective. In this setting, the subject is located inside a tent/room in which the ambient O_2_ concertation is kept at 13% (inducing a SaO_2_ of 82–85%) and he/she wears conventional nasal cannulas to administer a short bolus (e.g., 15 s) of pure O_2_ to induce reoxygenation [166,167]. By adjusting the gas flow of the O_2_ bolus, the setting allows achieving SaO_2_ cycling within the desired limits (e.g., 95–85%). A similar hypoxic time after the O_2_ bolus allows for applying the challenge at frequencies around 30 cycle/h to realistically mimic SA. The most practical advantage of this setting is that the subject can feel more comfortable since instead of being attached to a mask and tubing, he/she simply wears nasal prongs hence feeling less constricted and facilitating free movement within the hypoxic tent/room, which is of particular interest if the model is to be applied daily during sleep for several weeks [149].

Human models based on applying a SF for studying the end-organ effects of SA are limited. Although there is ample literature experimentally studying the effects of sleep alterations such as circadian cycle shift, sleep deprivation, or sleep restriction, there are few settings of human models in which SF is applied with a pattern realistically mimicking SA. SF has been applied unspecifically [168] or specifically interrupting SWS or REM sleep [169], which are paradigms relatively different from those experienced by SA patients. The most realistic SA model of SF in humans consists of applying SF at a frequency of 30 events/hour throughout the whole night irrespective of sleep stage. SF fragmentation—EEG microarousals > 3 s—was achieved by auditory and mechanical vibration stimuli in anticipation of habituation that may occur with a single repeated auditory stimulus type. This model was applied just for two sequential nights to study the effects of SF on cognitive processing, glucose metabolism or periodic leg motor activity [170,171,172].

### 4.2. Experimental SA Models Based on Patients

**Figure 4:**
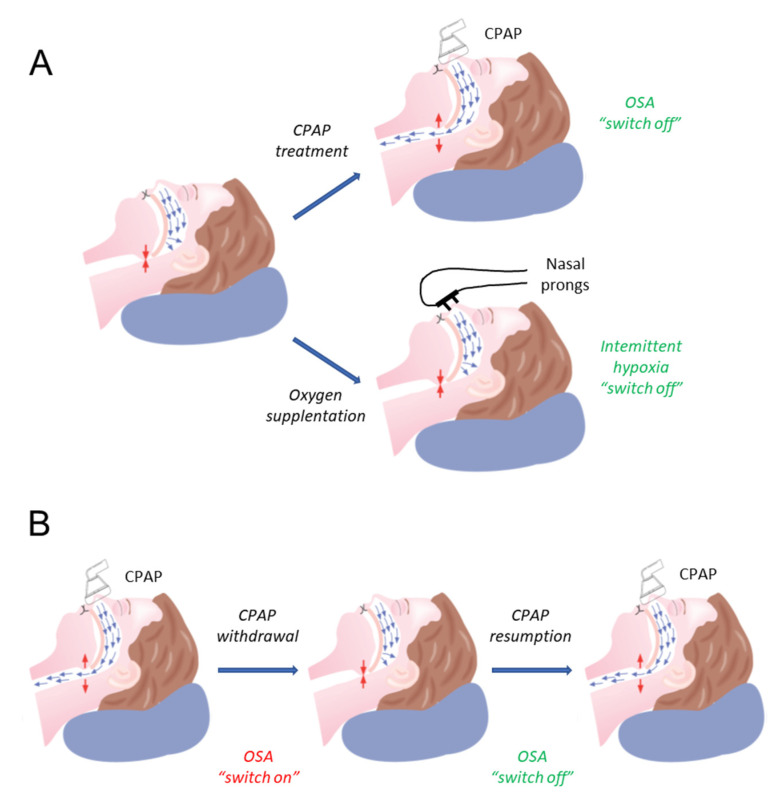
Experimental patient models to study the mechanisms involved in obstructive sleep apnea (OSA) consequences. (**A**) Model of supplementary nocturnal oxygen therapy. Patients with OSA experiencing upper airway collapse (with associated intermittent hypoxemia, sleep fragmentation and increased intrathoracic pressure swings) can be subjected either to continuous positive airway pressure (CPAP) therapy (equivalent to “switching off” OSA if CPAP is effective) or to supplementary oxygen therapy to avoid only the recurrent oxygen arterial desaturations induced by OSA. (**B**) Model of CPAP withdrawal. Patients effectively treated with CPAP (therefore not experiencing OSA challenges) are modelling “normal” subjects as compared with the same patient after CPAP withdrawal, an intervention equivalent to “switching on” OSA. Resumption of CPAP after withdrawal recovers the baseline condition. Comparison of patient status after “switching on and off” OSA provides information on the mechanisms involved in the consequences of this sleep-breathing disorder. Reprinted with permission from Ref. [173]. 2021. European Respiratory Society.

Models of SA based on subjecting healthy volunteers to challenges mimicking the disease is the usual way of conceiving experimental human models when studying the pathophysiology of the disease. However, another type of human SA model may provide very useful mechanistic information on the consequences of SA. This approach moves the mechanistic research from the physiology lab to the clinics and is based on subjecting patients with SA to simple clinical interventions and evaluating the changes elicited [173]. One possible intervention (Figure 4) is to compare the mid and short-term changes (e.g., cardiovascular, metabolic, neurocognitive) experienced by a recently diagnosed patient after being treated with either CPAP or oxygen. In the first case, if apneas and hypopneas are avoided with CPAP, all the noxious stimuli experienced by the patient would disappear. By contrast, in the second case, the events of intermittent hypoxia would disappear but intermittent hypercapnia, arousals, and increased inspiratory efforts would remain. Whereas a comparison of the differential effects of administering CPAP and oxygen has been extensively carried out from a therapeutic perspective [174,175], few studies have used this approach for mechanistic research purposes [176,177].

Another human model based on SA patients is CPAP withdrawal (Figure 4). In this case, the comparison is carried out between the status of a patient effectively treated with CPAP with his/her status after short- or mid-term CPAP withdrawal [178]. In this setting, a patient who could be considered a “healthy subject” (with no SA events because of effective CPAP) is suddenly transformed into a “new” SA patient. The CPAP withdrawal paradigm has been well characterized and has provided interesting results on the end-organ consequences of SA [178,179,180,181].

Patient-based experimental models to study the mechanisms involved in the consequences of SA (e.g., oxygen therapy and CPAP withdrawal) have some limitations but are very easy to implement in the clinical setting and therefore are potentially applicable to a high number of patients focusing on selected SA phenotypes [145,179,180,181,182,183,184,185,186] and biomarkers [187]. The information provided by these models could be enhanced by incorporating the techniques of genomics and proteomics and the current and future tools provided by big data [188] and artificial intelligence [189].

## 5. Conclusions

**Figure 5:**
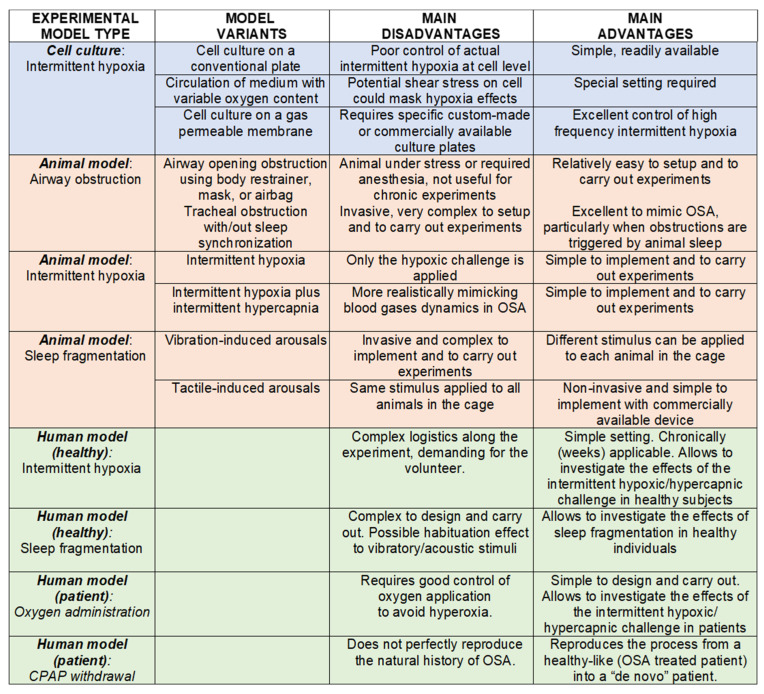
Experimental models to investigate the end-organ consequences of SA.

The experimental research in SA models at the cell, animal and human levels has been so far very productive, with advantages and disadvantages summarized in Figure 5. Regarding 2D cell culture, the application of intermittent hypoxia/hypercapnia at cycling rates mimicking severe apneas is currently possible by using optimized settings. Future developments should allow applying controlled gas concentration cycles to cells seeded into 3D scaffolds mimicking in vivo conditions in cells other than epithelial and endothelial cells. Current animal models permit subjecting the animals to well-controlled and realistic stimuli of upper airway obstruction, intermittent hypoxia (hypercapnia and sleep fragmentation). A possible future improvement could be achieved by applying SA stimuli following a more realistic experimental design such as better controlling age, sex, genetic variability, ambient temperature, gut microbiota, and social interaction. Finally, human experimental models, particularly in SA patients, based on oxygen supplementation and CPAP withdrawal are promising for investigating mechanisms of end-organ morbidity, particularly if combined with advanced integrative tools such as big data and artificial intelligence.

## Data Availability

Not applicable.
